# Effect of Residual Deformation Energy and Critical Heating Rate on Cubic Texture and Grain Growth Behavior of Severely Deformed Aluminum Foil

**DOI:** 10.3390/ma15041395

**Published:** 2022-02-14

**Authors:** Yunlei Wang, Liping Ren, Qi Liu, Yu Cao, Guangjie Huang

**Affiliations:** 1 College of Materials Science and Engineering, Chongqing University of Arts and Sciences, Chongqing 402160, China; renlp315@163.com; 2 National Instrument Functional Materials Engineering and Technology Research Center, Chongqing Materials Research Institute Co. Ltd., Chongqing 400707, China; liuqicqu@yeah.net; 3 College of Materials Science and Engineering, Chongqing University, Chongqing 400044, China; caoyughost@yeah.net (Y.C.); gjhcqu@gmail.com (G.H.)

**Keywords:** residual deformation energy, critical heating rate, grain growth behavior

## Abstract

To clarify the microstructure, grain size, and recrystallization behavior during different annealing processes with controlled heating rates, the aim of this study was to investigate the effect of residual deformation energy after cold rolling and critical heating rate on cubic texture components, and grain growth behavior of aluminum plate, which was subjected to severe deformation. The experimental results revealed that the stored energy can be inferred from a calculation that fast annealing (FA) for 30 s was 2.2 times as large as slow annealing (SA) at 320 °C, which provided the driving force for grain growth during subsequent heating and resulted in a significant coarsening of grains in the FA process. In contrast, the intensity of cubic texture in SA was significantly higher than that in the FA process. A critical heating rate of 50 °C/min had been obtained to produce a homogeneous microstructure and strong cubic texture during the annealing processes with controlled heating rates and was verified by experiment. The relationship of Δ*η*_sur_ > 0.02*η*_b_ was as a criterion used to determine whether abnormal grain growth happened in aluminum foil, while the grain size exceeded the thickness of aluminum foil by examined calculation.

## 1. Introduction

In previous works [1,2,3,4,5], researchers had carried out many studies on the recrystallization, grain size change, and grain misorientation distribution of high-purity aluminum foil in conventional annealing processes, which formed a relatively mature theoretical system. However, in the preparation process of high-purity aluminum foil, there were few reports on optimizing the annealing system by controlling the heating rate, and this research had a certain innovation and practical application value.

The microstructures and properties were the primary concerns in the research of aluminum alloy during the cold rolling and annealing process [6,7,8,9,10,11]. Especially, residual deformation energy storage was very important when affecting recrystallization and grain growth after the annealing processes. Aluminum was used widely in foil-form for high voltage electrolytic capacitor anode aluminum foil, and packaging at thicknesses of just over 0.11 μm to around 150 μm [12,13]. In the manufacturing industry, the biggest usage of high purity aluminum foil was to produce aluminum electrolytic capacitors. This kind of aluminum electrolytic capacitor had advantages of large capacitance, high working voltage, and low price. Especially in recent years, the performance of aluminum foil had improved to a certain extent, and the application scope was gradually expanded. The high level of cold reductions of aluminum foil was always subjected to final annealing and obtained a large scale of cube components [14,15,16]. However, abnormal grain growth behavior was obviously and commonly observed in annealing aluminum foil. The cube texture played a significant role and acted as a typical recrystallization texture to control the microstructures, which seriously affected the capacitance of the electrolytic capacitor, based on the influence of the cube texture on qualities of aluminum foil, the evolution of microstructures, and textures that were investigated in the FA process with different heating rates. In previous studies, the influence of Zn and/or Ag additions on microstructure and properties of Al-Mg based alloys was studied [17], which suggested that the microstructure and properties evolution law in different Zn and/or Ag additions exists, while Ferry and co-worker [18] studied the influence of rapid heating rates on recrystallization nucleation and final grain size in particle-containing aluminum alloys. However, the studies of the effect of different heating rates on cube texture and abnormal grain growth during the FA process were rarely reported for high-voltage anode aluminum foil.

In order to obtain a uniform grain structure and strong cube texture component in aluminum foil using the field of high voltage electrolytic capacitor, in this paper, we adopted the samples with the furnace heating to annealing for finished products. Through the experiment with fast annealing and slow annealing processes to simulate the heating rate controlling product annealing process, the effects of different heating rates on the microstructure and texture component evolution of high purity aluminum foil were discussed carefully; to seek a suitable heating rate to replace the conventional annealing process, and optimize annealing processes and save production costs.

## 2. Experimental

### 2.1. Materials

The chemical composition of as-received aluminum plate is mainly composed of 99.995% aluminum (Al), 0.001% iron (Fe), 0.0012% copper (Cu), 0.0016% magnesium (Mg), 0.0007% silicon (Si), 0.0002% manganese (Mn), 0.0001% nickel (Ni), 0.0003% zinc (Zn) and 0.0001% titanium (Ti). Table 1 shows the complete chemical composition of aluminum plate.

The initial materials of aluminum plates were the three-layered liquid electrolysis, hot-rolled, high-purity aluminum plates (purity over 99.99%), which were provided by Southwest Aluminum (Group) Co., Ltd. (Chongqing, China). The chemical component was detected using a PDA-6000 photoelectric spectrometer (Shimadzu, Chengdu, China). The high purity only includes trace impurity elements, and these impurity elements were existing in the aluminum matrix in the form of a solid solution. Therefore, the effect of the impurity, second phase on recrystallization, and grain growth could be ignored.

### 2.2. Sample Preparation

**Figure 1:**
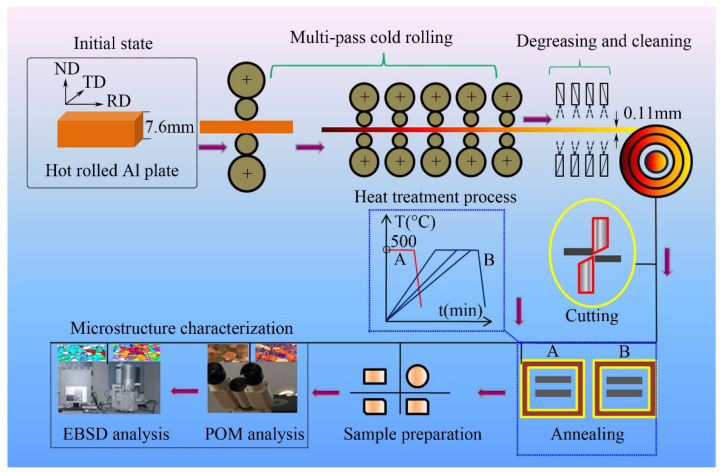
The schematic of processing route and sample characterization.

The initial aluminum plate was hot rolled and its thickness was 7.6 mm, then, it was subjected to severe and multi-pass cold rolling to a final thickness of 0.11 mm. The deformation degree was over 98% (as shown in Figure 1), the final samples need to degrease and clear by degreaser or weak acid. The samples were cut into various shapes to meet the need of the subsequent experiment.

### 2.3. Characterization

The annealed samples were anodic coated and electropolished, the microstructure and texture were observed by Axiovert 40MAT polarized optical microscopy (POM) (Zeiss, Oberkochen, Germany). Misorientation angle and grain boundary characteristics were analyzed by FEI NOVA 400 scanning electron microscopy (SEM) (FEI, Hillsboro, America) equipped with an electron backscattered diffraction probe (That’s EBSD technology) on the RD/ND or RD/TD planes. The samples were first cut into 10 mm × 5 mm × 0.11 mm. The microstructures of the as-received aluminum plate and its foil were characterized by backscattered electron (BSE) imaging with a Zeiss (Oberkochen, Germany) Supra 55 SEM, the testing data was processed using the software of channel 5, a significant texture analysis typically over 2500 grains had to be evaluated. Specimens of detected areas were 300 µm × 500 µm, and the step size was set as 0.5 µm. The EBSD mapping was prepared by mechanical polishing down to colloidal Al_2_O_3_, and samples for BSE observation were slightly etched by Keller reagent (consisting of 2 mL HF, 3 mL HCl, 5 mL HNO_3_, and 190 mL H_2_O) after mechanical polishing.

In this experimental annealing process, the two kinds of annealing methods were designed in our experiment routes, which were the slow annealing (SA) and fast annealing (FA) process, which was mentioned in my previous research work [19,20]. The details of SA are that the samples were heated from a room temperature to target temperature with a low heating rate (approximately 180 °C/h) and kept for 1 h; the FA is that the samples were kept at the target temperature for 30 s, 45 s, 1 h or 2 h, etc.

## 3. Results and Discussion

### 3.1. Grain Growth Rate of Fast Annealing and Slow Annealing Process

**Figure 2:**
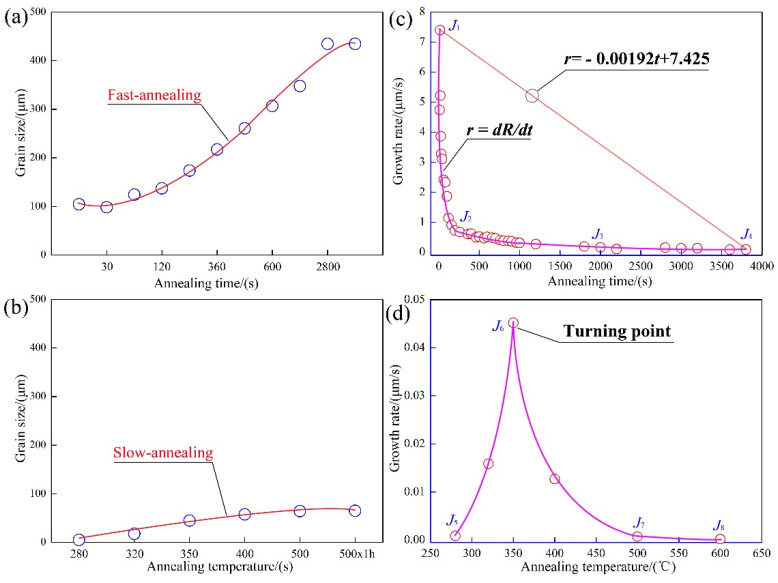
Grain size and grain growth rate of (**a**) fast annealing; (**b**) slow annealing; (**c**,**d**) grain growth rate corresponding to (**a**,**b**).

The relationship between recrystallized grain size and grain growth rate accompanied by annealing time and annealing temperature is shown in Figure 2. In the FA process, the grain growth rate curve can be divided into three sections, the first section of J_1_-J_2_ (Jn represents the curve of grain growth rate) was a parabolic curve with grain growth, while the second section of J_2_-J_3_ appeared, and it was a diagonal line, then, the third section of J_3_-J_4_, which was approximately horizontal and linear. J_1_-J_2_-J_3_-J_4_ was the grain growth curve (solid line) in the experiment, and the formula of grain growth rate was written as follows [21]:

(1) $$v=\frac{dR}{dt}$$

where *R* is the grain size, *t* is the annealing time when the grain size is *R*, and *v* is the grain growth rate, i.e., the ratio is between Δ*d* and Δ*t*.

The grain growth rate of the J_1_-J_2_ section was a curve, while the grain growth rate decreased with the annealing time increasing, which meant that the grain size grew rapidly in a parabolic shape in the early stage of annealing, but the grain growth rate in the later period was getting smaller and smaller. The grain growth rate of J_2_-J_3_ section was less than 0.5 μm/s, which indicated that the grain growth was more slow. The J_3_-J_4_ section was approximately a horizontal straight line, which indicated that the grain growth rate was zero and the grain tended to be basically stable. At this time, the corresponding annealing time was about 3600 s, which also meant that the grain cannot grow up without limited time, the grain size tended to be stable.

It can be seen that the grain growth rate reached the maximum value at the annealing temperature of 350 °C. After recrystallization, the grain growth rate was slow and basically stable. The final grain size was less than 100 μm, which did not exceed the thickness of aluminum foil. The samples were annealed at 500 °C, and 500 °C and held for 1 h, the grain size did not change significantly, and the grain did not continue to grow, which was still at a certain size of 60 μm.

In a word, when the grain size exceeded the critical size at the completion of recrystallization, the grain growth rate was slowed down and eventually approached zero. However, in the FA process, when the grain size exceeded the thickness of aluminum foil, abnormal grain growth behavior was observed, then the large grain size even reached the millimeter level.

### 3.2. Texture Component of Fast Annealing and Slow Annealing Process

**Figure 3:**
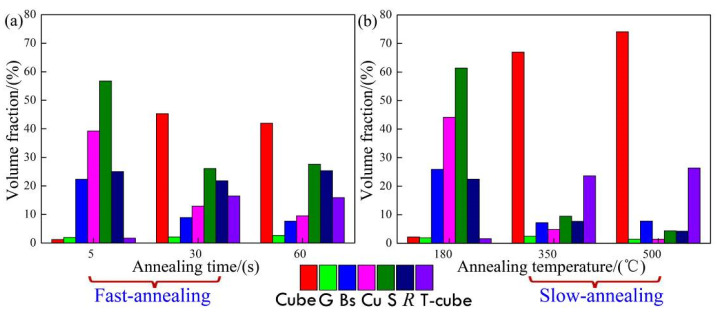
Typical texture volume fraction of aluminum foil in different annealing processes (**a**) fast annealing; (**b**) slow annealing.

The changing trend of various textures was observed in FA and SA processes. Figure 3 lists the statistical distribution of the seven typical textures volume fractions in two annealing processes. It can be seen from Figure 3a,b that the aluminum foil was finally dominated by cube texture components of {001}<100>, the cube texture volume fraction was 45% in the FA process with an annealing time of 60 s, while the cube texture content was 78% in the SA process with an annealing temperature of 500 °C. Where it reached the maximum, it was obviously higher than the cubic texture content after the FA process, which indicated that SA was beneficial to increase the cubic texture content in aluminum foil.

### 3.3. Recrystallization Behavior of Fast Annealing and Slow Annealing Process

**Figure 4:**
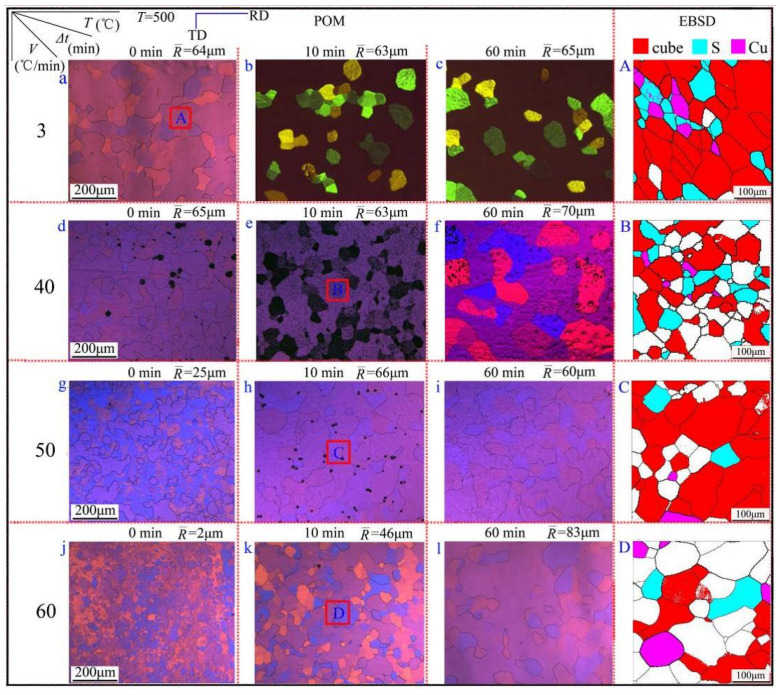
Microstructures of aluminum foil during the annealing process with different heating rates (**a**–**l**) polarized microstructures; (A–D) EBSD maps corresponding to (**a**–**l**).

In order to find a suitable heating rate in the SA process, it was combined with the recrystallization characteristics of aluminum foil; the annealed microstructure of four heating rates (*V* = 3, 40, 50, 60 °C/min) was observed and is given in Figure 4. When the aluminum foil was heated to 500 °C with a heating rate of 3 °C/min, the aluminum foil underwent complete recrystallization. With the extension of holding time to 10 min and 60 min, the grain size did not seem to change much more, which indicated that the storage energy was fully released. While the recrystallization was completed, it made the secondary recrystallization lose power temporarily, thus, the grain size did not change much (as shown in Figure 4a–c). When the annealing heating rate was 50 °C/min, it could be seen that the grain size grew significantly with the holding time extending from 0 min to 10 min. There was no significant change in grain size between the holding time of 10 min and 60 min, which indicated that the deformation storage energy was not released completely at the beginning. With the holding time extension, it had enough driving force to make the grain grow sequentially [15].

The storage energy was released after the holding time of 10 min. The grain size did not change much after a holding time of 60 min (as shown in Figure 4j–l). In order to further confirm whether the 50 °C/min was the best heating rate, the annealing heating rate of 60 °C/min was studied subsequently. It can be seen from the results that the grain size changed greatly in the process of holding time from 10 min to 60 min, which was not the ideal heating rate.

In industrial production, the need to obtain an ideal organization structure at the same time as to save the production cost, thus, to sum up, in revealing the microstructure and cubic texture evolution law during FA and SA processes, and combining with the actual production process, it can be seen that the production processing of high purity aluminum foil is not recommended for the sample when the annealing temperature reached the target value. However, the sample annealing can be carried out by furnace heating (heating rate *V* = 50 °C/min, holding time Δt = 10 min), so it ensured the microstructure uniformity and obtained high volume fraction with cubic texture content, which had significant steps for guiding practical production.

### 3.4. Residual Deformation Energy Discussion of Fast Annealing and Slow Annealing Process

**Figure 5:**
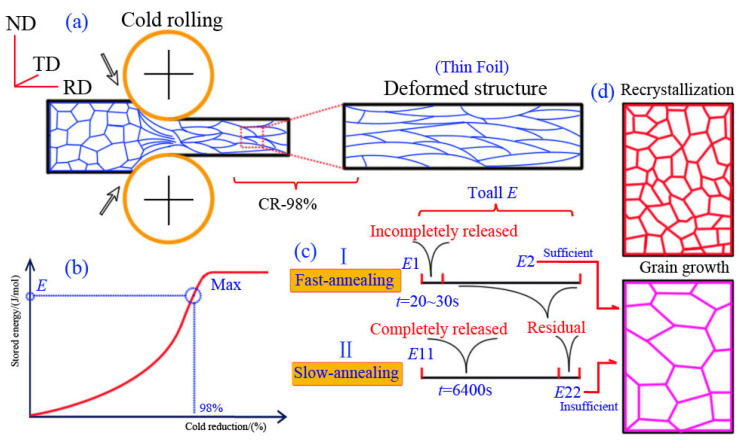
Schematic of deformation stored energy release of high purity aluminum foils during annealing (**a**) cold rolling; (**b**) stored energy versus cold reduction; (**c**) two typical annealing processes; (**d**) microstructure evolution.

To clarify the reasons for abnormal grain growth behavior in the FA process, and why it did not happen in the SA process, the release of deformation storage energy in the process of recovery and recrystallization was studied qualitatively (as shown in Figure 5). According to the experimental results, complete recrystallization had taken place in the sample during FA at 20~30 s, and the recrystallization was completed in a relatively short time without considering other energy loss. It was considered that the deformation energy storage will be consumed by the recrystallization of E_1_, which indicated that the recrystallized sample with 30 s still retained enough deformation energy storage, and the remaining deformation energy storage was E_2_, as shown in Figure 5c. In the SA process, the recrystallization was completed at 320 °C, which took about 6400 s, due to the longer annealing time—most of the energy storage had been fully released in the recrystallization process, and the deformation storage energy of E_11_ was consumed. Residual energy storage E_22_ was insufficient to provide the driving force for later grain growth, rather, it combined with the result of what was increasing the annealing temperature and holding time—the grain size was not large, which can be confirmed, and that the residual deformation energy storage was less after full recrystallization. The driving force for grain growth was insufficient, which resulted in the grain size remaining within a certain range and not growing.

**Figure 6:**
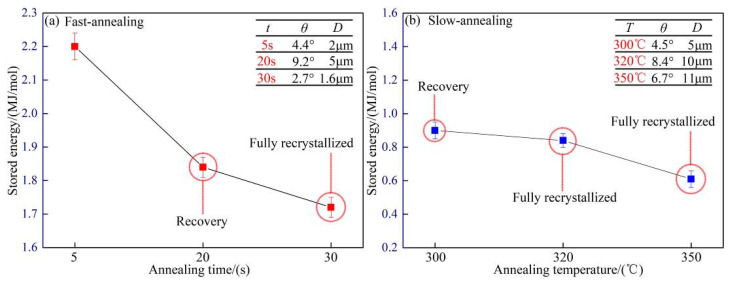
Semi-quantitative calculation of stored energy during annealing (**a**) fast annealing; (**b**) slow annealing.

To further verify that there was still a large amount of deformation energy storage after 30 s full recrystallization during the FA process, and while recrystallization was at 350 °C for the SA process, the deformation energy storage was released basically. Humphreys et al. [22] gave the relationship between storage energy, misorientation angle, and sub-crystal size, which can calculate the size of *E_D_* of energy storage semi-quantitatively.

(2) $$E_{D}=\frac{3\eta_{b}\theta }{D\theta_{m}}\left(1-\ln\frac{\theta }{\theta_{m}}\right)\approx \frac{K\theta }{D}$$

where *η*_b_ is the interface energy of low angle grain boundary, *θ* is the misorientation angle, *θ*_m_ is the misorientation angle when grain boundary changes to a large angle grain boundary, *D* is the subgrain size, and *K* is a constant. After calculation for the data (as shown in Figure 6), the storage energy *E*_*D*1_ was 1.84*K* after full recrystallization for 30 s in the FA process, and *E*_*D*2_ was 0.84*K* after annealing at 320 °C in the SA process. It can be seen that *E*_*D*1_: *E*_*D*2_ = 2.2, that said, the former energy storage was 2.2 times to the latter, which indicated that the high-purity aluminum foil with 98% deformation had completed recrystallization in FA for 30 s, and the deformation storage energy was not fully released; however it also retained a large amount of deformation storage, which provided the driving force for secondary recrystallization. However, after a long time of recovery and recrystallization during annealing at 320 °C, the deformation storage energy was basically released, and the secondary recrystallization power was lost.

**Figure 7:**
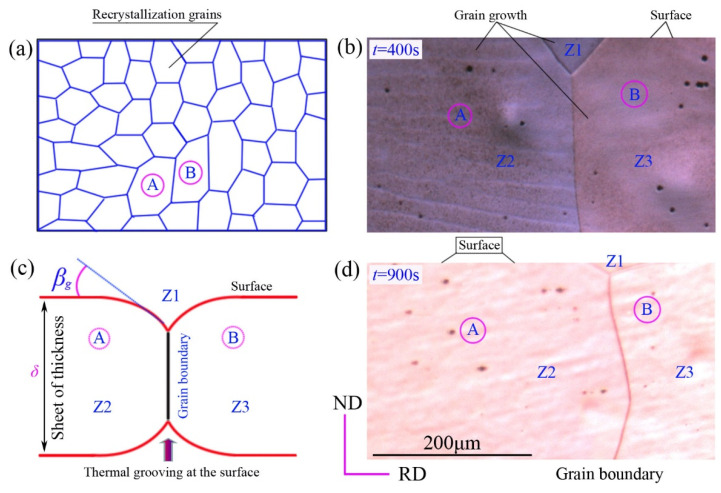
Schematic of thermal grooving of high purity aluminum foils during annealing (**a**) recrystallization; (**b**,**c**) grain growth at *t* = 400 s and *t* = 900 s; (**d**) model of thermal grooving at the surface.

In a thin aluminum plate or myopic film material, the grain growth after recrystallization seemed to be different from that in a thick plate. The main reason was that when the grain size exceeded the thickness of aluminum foil, the grain was completely long and transparent along the direction of plate thickness, which showed that the share of the surface was obviously greater than that in the interface, so the surface energy of the grain was greater than its interface energy. During the annealing process, the grains grew along the direction of aluminum foil thickness, which was prone to thermal erosion grooves, as shown in Figure 7.

In our experiment, it was found that the thermal erosion groove appeared at the time of 400 s during the FA process, while the annealing time extended to 900 s, and the thermal erosion groove (Z_1_ zone) moved with the grain boundary migration. As shown in Figure 7a, the two recrystallized grains A and B had the size advantage, and with the extension of annealing time, grains A and B still grew rapidly even when the other surrounding grains’ growth was blocked. Along the direction of aluminum foil thickness, the size of grains A and B exceeded the thickness of aluminum foil and became long permeable. At the grain boundary, where grain A and B contacted and closed to the aluminum foil surface, thermal erosion grooves (Z_1_ zone) appeared, and that was divided into three zones of Z_1_-Z_2_-Z_3_.

It was found that the deformation storage energy and surface energy were the main driving forces to control the grain growth of aluminum foil during the whole annealing process. On one hand, the driving force controlling grain recrystallization and grain growth before grain size exceeded the aluminum foil thickness, was systematic deformation storage; On the other hand, after the grain size exceeded aluminum foil thickness, the driving force controlling grain growth was the difference of surface energy between adjacent grains.

Mullins et al. [23] found in the study on the variation of thermal erosion groove with grain boundary migration during annealing for thin sheet materials, and the growth conditions of abnormal grains were written as follows that:

(3) $$2\Delta \eta_{sur}>\frac{\eta_{b}^{2}}{\eta_{sur}}$$

Under normal conditions, *η*_sur_ is about three times as large as *η*_b_, that is to say, *η*_sur_ = 3*η*_b_, when Δ*η*_sur_ > 0.02*η*_b_, which is substituted into Equation (3), the abnormal grain growth will occur in this situation. We know that the *η*_sur_ represents surface energy, *η*_b_ represents grain boundary energy, Δ*η*_sur_ represents the surface energy difference between adjacent grains, *δ* represents thin plate thickness, and *M* represents the grain boundary mobility. The given formula to driving force of grain boundary migration is *P*_s_ = 2Δ*η*_sur_*/δ*, and the napping force of thermal erosion groove is *P*_T_ = *η*_b_^2^*/δη*_sur_. Therefore, the abnormal grains must overcome the napping of the thermal erosion groove to grow [12], and the grain boundary migration rate is written as:

(4) $$v=M\left(P_{s}-P_{T}\right)=\frac{M}{\delta }\left(2\Delta \eta_{sur}-\frac{\eta_{b}^{2}}{\eta_{sur}}\right)$$

By modifying and simplifying the formula, it can be concluded that Δ*η*_sur_ > 0.02*η*_b_, and can be used as a criterion of abnormal grain growth in thin plate materials. Combined with Figure 3, it can be seen that the grains of (001) and (111) face were the main ones after aluminum foil recrystallization, and the surface energy of the crystal face as the surface was given by Zhang [24]. The surface energy surface density of the face-centered cubic metal aluminum was represented by *η*_sur_.

The related parameters of pure aluminum *η*_(001)sur_ = 898 mJ/m^2^, *η*_(111)sur_ = 619 mJ/m^2^, and calculated the Δ*η*_sur_ = 279 mJ/m^2^, 0.02*η*_b_ = 200 mJ/m^2^, it can be seen that Δ*η*_sur_ > 0.02*η*_b_ satisfied the condition. In this experiment, abnormal grain growth was found in the FA process. It can be seen that when the grain size exceeded the thickness of aluminum foil, the abnormal grain growth behavior can be explained by the theory of surface energy controlling grain growth.

## 4. Conclusions

This paper reported the microstructure, texture component, and recrystallization behavior evolution of high-level deformation aluminum plates with different heating rates during the annealing process, and was conducted using POM and EBSD technology. The results were summarized as follows:

(1) Residual deformation energy storage can be inferred from the calculation of FA for 30 s and was 2.2 times as large as SA at 320 °C, which provided the driving force for grain growth during subsequent heating, which resulted in a significant coarsening of grains through FA. In contrast, the intensity of the cubic texture after SA was significantly higher than that after the FA process.
(2) The microstructure and texture component evolution of high purity aluminum foil with severe deformation were studied during different annealing processes by controlled heating rates; the critical heating rate obtained was 50 °C/min, and it could gain a homogeneous microstructure and strong cubic texture.
(3) The grain growth was mainly controlled by surface energy when the grain size exceeded the thickness of aluminum foil. In this situation, this can be used as a criterion to determine whether abnormal grain growth happened in aluminum foil with the relationship of Δ*η*_sur_ > 0.02*η*_b_.

## Figures and Tables

**Table 1:** Chemical composition of initial aluminum plates (mass content in ppm).

Chemical Elements	Al	Fe	Cu	Mg	Si	Mn	Ni	Zn	Ti
Mass content	Bal.	10	12	16	7	2	1	3	1

## Data Availability

Not applicable.
